# A Conjugative MDR pMG1-Like Plasmid Carrying the *lsa*(E) Gene of *Enterococcus faecium* With Potential Transmission to *Staphylococcus aureus*

**DOI:** 10.3389/fmicb.2021.667415

**Published:** 2021-06-04

**Authors:** Xiao-Mei Yan, Jing Wang, Xiao-Xia Tao, Hong-Bing Jia, Fan-Liang Meng, Hui Yang, Yuan-Hai You, Bo Zheng, Yuan Hu, Xiao-Xia Bu, Jian-Zhong Zhang

**Affiliations:** ^1^State Key Laboratory of Infectious Disease Prevention and Control, Collaborative Innovation Center for Diagnosis and Treatment of Infectious Diseases, National Institute for Communicable Disease Control and Prevention, Chinese Center for Disease Control and Prevention, Beijing, China; ^2^Department of Clinical Diagnosis, China-Japan Friendship Hospital, Beijing, China; ^3^Institute of Clinical Pharmacology, Peking University First Hospital, Beijing, China

**Keywords:** *Enterococcus faecium*, conjugative plasmid, *lsa*(E), *Staphylococcus aureus*, quinupristin/dalfopristin

## Abstract

*lsa*(E) is a pleuromutilin, lincosamide, and streptogramin A (PLSA phenotype) resistance gene that was first described in *S. aureus* and was thought to have been transferred from *Enterococcus* sp. This study aimed to elucidate the prevalence of the *lsa*(E) gene among *E. faecium* isolates at a tertiary teaching hospital and to evaluate the transferability of the *lsa*(E) gene from *E. faecium* to *S. aureus in vitro*. A total of 96 *E. faecium* strains isolated from one hospital in Beijing in 2013 were analysed for quinupristin-dalfopristin (QDA) resistance genes, and multilocus sequence typing (MLST) was performed. The transferability of QDA resistance between ten *E. faecium* strains and four *S. aureus* strains was determined by filter mating. Genome sequencing of the transconjugant was performed. A total of 46 *E. faecium* isolates (46/96, 47.92%) tested positive for *lsa*(E), while two isolates (2/96, 2.08%) tested positive for *lsa*(A). Thirty-six *lsa*(E)-positive strains (36/46, 78.3%) belonged to ST78. Among 40 mating tests, *lsa*(E) was successfully transferred through one conjugation at a frequency of 1.125 × 10^–7^ transconjugants per donor. The QDA resistance of the transconjugant N7435-R3645 was expressed at a higher level (MIC = 16 mg/L) than that of the parent *S. aureus* strain (MIC = 0.38 mg/L). Next-generation sequencing (NGS) analysis of the transconjugant N7435-R3645 showed that the complete sequence of the *lsa*(E)-carrying plasmid pN7435-R3645 had a size of 92,396 bp and a G + C content of 33% (accession no. MT022086). The genetic map of pN7435-R3645 had high nucleotide similarity and shared the main open reading frame (ORF) features with two plasmids: *E. faecium* pMG1 (AB206333.1) and *E. faecium* LS170308 (CP025078.1). The *rep* gene of pN7435-R3645 showed 100% identity with that of pMG1, although it did not belong to the *rep*1-19 family but instead a unique *rep* family. Multiple antibiotic resistance genes, including *lsa*(E), *aadE* and *lnu*(B), *erm*(B), *ant6*-Ia, and *lnu*(B), were present on the plasmid. In conclusion, an *lsa*(E)-carrying plasmid that can be transferred by conjugation from *E. faecium* to *S. aureus in vitro* was identified. This multidrug resistance (MDR) pMG1-like plasmid may act as a vector in the dissemination of antimicrobial resistance among species.

## Introduction

Enterococci and *Staphylococcus aureus* are well-documented opportunistic pathogens. Due to the emergence of antimicrobial resistance as a result of antibiotic overuse, a great concern is infection by methicillin-resistant *Staphylococcus aureus* (MRSA) and vancomycin-resistant *Enterococcus* species (VRE), which can lead to increased treatment failure and higher mortality rates (Blot et al., 2002; Yaw et al., 2014). Antibiotic resistance in *S. aureus* can emerge through point mutations or horizontal transfer of mobile genetic elements (MGEs). Genetic exchange of genes coding for antibiotic resistance between enterococci and *S. aureus* has been reported for genes such as the vancomycin resistance gene *vanA* (Weigel, 2003), the tetracycline resistance gene *tetM* (Leon-Sampedro et al., 2016), the trimethoprim resistance gene *dfrK* (Lopez et al., 2012), the multiresistance gene *cfr* (Liu et al., 2012) and the macrolide resistance gene *erm*(B) (Wan et al., 2016).

Quinupristin-dalfopristin (QDA) is a semisynthetic 70:30 mixture of streptogramin A and B and is used mainly for the treatment of glycopeptide-resistant *Enterococcus faecium* (GRE) and MRSA infections. The two mixture components act synergistically on the bacterial 50S ribosomal subunit, inhibiting protein synthesis. Resistance to streptogramin B does not confer resistance to QDA, while resistance to streptogramin A does (Hancock, 2005). Resistance to streptogramin A-type antibiotics can be caused by different mechanisms, such as the acetyltransferase Vat (Allignet et al., 1993), the ABC transporters Vga (Allignet et al., 1992, 1998; Kadlec and Schwarz, 2009; Schwendener and Perreten, 2011) and Lsa (Wendlandt et al., 2013b), and the methyltransferase Cfr (Long et al., 2006).

The *lsa*(E) gene was first described in three *S. aureus* strains of human origin, namely, one MRSA ST398-t011 strain and two methicillin-susceptible *S. aureus* (MSSA) ST9-t337 strains, and encodes an ABC transporter of unknown function (Wendlandt et al., 2013b). The *lsa*(E) gene was identified as a macrolide-lincosamide-streptogramin (MLS) resistance gene and was speculated to have been transferred from *Enterococcus* (Wendlandt et al., 2013b). The *lsa*(E) gene has been described not only in *S. aureus* but also in coagulase-negative staphylococci (CoNS) and other species, such as *Erysipelothrix rhusiopathiae*, *Streptococcus suis*, and *Streptococcus agalactiae* (Montilla et al., 2014; Wendlandt et al., 2015; Zhang et al., 2015; Huang et al., 2016). It is most often located in a multiresistance region in chromosomal DNA (Wendlandt et al., 2013b, 2014, 2015; Sarrou et al., 2016; Deng et al., 2017) and is sometimes detected on plasmids (Li et al., 2013; Wendlandt et al., 2013a).

We previously demonstrated that 98% (44/45) of QDA-resistant *S. aureus* isolates sampled from slaughter pigs in northeastern China harboured *lsa*(E) (Yan et al., 2014). Genome sequencing of the *lsa*(E)-positive strains revealed that the transposon with the *lsa*(E) gene cluster showed similarity to the plasmid pEF418 of *E. faecalis* and the plasmid pXD4 of *E. faecium* (Yan et al., 2016). However, limited information is known about the presence of the *lsa*(E) gene in *E. faecium* strains isolated from inpatients in China and the transferability of the *lsa*(E) gene between *E. faecium* and *S. aureus*.

The objective of this study was to elucidate the prevalence of the *lsa*(E) gene among *E. faecium* strains isolated at a tertiary teaching hospital and to evaluate the transferability of the *lsa*(E) gene from *E. faecium* to *S. aureus in vitro*.

## Materials and Methods

### Bacterial Isolates

A total of 96 *E. faecium* strains isolated from one hospital in Beijing in 2013 were analysed in the present study (Supplementary Table 1). The isolates were identified as *E. faecium* using a Vitek-2 microbiology analyser (bioMérieux, Marcy l’Etoile, France).

### Antimicrobial Susceptibility Testing and QDA Resistance Gene Detection

The susceptibility to 13 antimicrobial agents—ampicillin, penicillin, erythromycin, ciprofloxacin, levofloxacin, nitrofurantoin, tetracycline, vancomycin, linezolid, quinupristin/dalfopristin, tigecycline, high-level gentamicin and streptomycin—was tested with a Vitek-2 microbiology analyser according to the manufacturer’s instructions. QDA resistance was reconfirmed by Etest (bioMérieux SA, Marcy l’Etoile, France). The minimum inhibitory concentrations (MICs) for all the antimicrobials were interpreted using Clinical and Laboratory Standards Institute (CLSI) criteria [Clinical and Laboratory Standards Institute (CLSI), 2021].

All the isolates were investigated for the QDA resistance genes *lsa*(A), *lsa*(C), *lsa*(E), *vat*D, *vat*E, *vatH*, and *vga*D by PCR, and *eat*(A) mutations, which are designated *eat*(A)v, were checked by sequencing (Supplementary Table 2).

### Multilocus Sequence Typing (MLST)

MLST of *E. faecium* isolates was performed by amplifying seven housekeeping genes—*adk*, *atpA*, *ddl*, *gyd*, *gdh*, *purK* and *pstS*—as described previously (Homan et al., 2002). The sequences were submitted to the MLST website for *E. faecium*^1^, and sequence types (STs) were assigned according to the allelic profiles. The clonal complex (CC) was analysed with goeBURST v1.2.1.

### Mating Experiments

The transferability of QDA resistance was determined by performing filter mating. Ten rifampin-susceptible *E. faecium* strains (9200, P9772, 5118, 6354, 6474, 3240, 4103, N7435, P2505 and P3814) harbouring *lsa*(E) were randomly selected as donors for the mating experiments. The recipients were four clinical *lsa*(E)-negative, rifampin- and methicillin-resistant *S. aureus* isolates (109, R3645, R3680, and 121) that were plasmid-free after plasmid extraction (Table 1). A donor:recipient ratio of 1:9 was used for the mating experiments (Tomita et al., 2002). Selection was performed on brain-heart infusion agar (BHI, OXOID LTD., Basingstoke, Hampshire, England) supplemented with 4 or 8 mg/L virginiamycin and 128 mg/L rifampicin. Rifampicin- and virginiamycin-resistant colonies of putative *S. aureus* transconjugants were isolated and identified by *lsa*(E) PCR. QDA was determined by Etest for the *lsa*(E)-positive transconjugant. The microdilution broth method was used to determine the MICs of 18 antimicrobial agents, namely, penicillin, cefoxitin, tetracycline, chloramphenicol, ciprofloxacin, gentamicin, rifampicin, vancomycin, nitrofurantoin, trimethoprim-sulphamethoxazole, erythromycin, teicoplanin, clindamycin, linezolid, tigecycline, mupirocin, fusidic acid, and daptomycin. The transfer frequency was expressed as the number of transconjugants per donor.

**TABLE 1:** Background of donor and recipient strains.

**Donor/Recipient**	**Species**	**Strain name**	**MLST**	***spa***	**Antibiotic resistance profile^*a*^**
Recipient	*S. aureus*	109	ST239	t1152	FOX-TC-GM-CI-EM-CM-RI
Recipient	*S. aureus*	R3645	ST239	t037	FOX-TC-GM-CI-EM-CM-RI
Recipient	*S. aureus*	R3680	ST239	t037	FOX-TC-GM-CI-EM-CM-RI
Recipient	*S. aureus*	121	ST239	t030	FOX-TC-GM-CI-EM-CM-RI
Donor	*E. faecium*	9200	ST747	–	
Donor	*E. faecium*	P9772	ST923	–	
Donor	*E. faecium*	5118	ST18	–	
Donor	*E. faecium*	6354	ST78	–	
Donor	*E. faecium*	6474	ST78	–	
Donor	*E. faecium*	3240	ST78	–	
Donor	*E. faecium*	4103	ST571	–	
Donor	*E. faecium*	N7435	ST18	–	
Donor	*E. faecium*	P2505	ST78	–	
Donor	*E. faecium*	P3814	ST78	–	

*^*a*^FOX, cefoxitin; TC, tetracycline; GM, gentamicin; CI, ciprofloxacin; EM, erythromycin; CM, clindamycin; RI, rifampin; QDA, quinupristin/dalfopristin.*

### *SmaI*- and *S1* Nuclease (*S1*)-Pulsed-Field Gel Electrophoresis (PFGE), Southern Blotting and Hybridisation Assays

Transconjugants were further confirmed by Southern blotting. *SmaI-* and *S1*-PFGE analyses were performed as described previously (Tomita et al., 2002; Yan et al., 2011). Southern blotting was performed using a DIG High Prime DNA labelling and Detection Starter Kit (Roche, Basel, Switzerland) according to the manufacturer’s instructions. The digoxigenin-labelled *lsa*(E)-specific probe was prepared using primers (forward 5′-ACAGCGAGTTGTTTCCTGCT-3′; and reverse 5′-GCACGTTTCATCGCTTTTGC-3′) that amplified a 410-bp region of the *lsa*(E) gene. After *S1*-PFGE, the DNA was transferred to a nylon membrane (Hybond N, Amersham, United Kingdom) that was hybridised with the prepared *lsa*(E)-specific probe. Detection was performed using an NBT/BCIP colour detection kit (Roche, Switzerland).

### Transconjugant Stability

The stability of the *lsa*(E)-carrying transconjugants was evaluated by daily serial passage on antibiotic-free blood agar. Colonies were tested daily for *lsa*(E) by PCR. The stability of the *lsa*(E)-carrying plasmid was also evaluated by growing on virginiamycin (4 and 8 mg/L) MH agar after storage at 4 and −80°C for 4 weeks.

### Plasmid Sequencing, Assembly and Annotation

The transconjugant N7435-R3645 genome (named with donor and recipient strains) was extracted using a commercial kit (Promega, Madison, United States). Genome sequencing was performed by using the Illumina HiSeq 4000 platform and PacBio RS II platform (10 kb insert library; Pacific Biosciences, Menlo Park, CA, United States) at the Beijing Genomics Institute (BGI, Shenzhen, China).

*De novo* assemblies and contig assembly for the plasmid pN7435-R3645 of transconjugant N7435-R3645 were performed using Soapdenovo 2.0. Open reading frames (ORFs) were predicted with GeneMarkS.^2^ The overlapping regions were found by BLASTing the sequences of the beginning and the end of the final contig. The closed plasmid was confirmed by PCR (JH-F 5′-CTCTACCAGATGGTTGGAGCA-3′; JH-R 5′-CCTACGATCACGGCACCAAT-3′) and Sanger sequencing of the resulting amplicons. The plasmid nucleotide sequences were compared with sequences in the GenBank database using BLASTN.^3^

### Nucleotide Sequence Accession Number

The sequence of the conjugated plasmid pN7435-R3645 was deposited in the DDBJ/EMBL/GenBank nucleotide sequence databases under accession number MT022086.

## Results

### Antimicrobial Susceptibility

For the *E. faecium* isolates, the resistance rates to ampicillin, penicillin, erythromycin, ciprofloxacin, levofloxacin, nitrofurantoin, gentamicin, streptomycin, tetracycline and vancomycin were 91.67, 92.71, 94.79, 92.71, 92.71, 75.00, 66.67 56.25, 31.25 and 20.83%, respectively. A low resistance rate was observed for linezolid (1.04%) and tigecycline (1.04%).

### Antimicrobial Resistance Genotype and Phenotype of QDA Resistance in *E. faecium* Strains

A total of 46 *E. faecium* isolates (46/96; 47.92%) tested positive for *lsa*(E), while two isolates (2/96; 2.08%) tested positive for *lsa*(A). The *eat*(A)v mutation (C1349T) was found in 41 of 96 *E. faecium* isolates. The *vatD, vatE, vatH, vgaD* and *lsa*(C) genes were not detected in any of the isolates. Four antibiotic resistance gene profiles were observed, namely, *lsa*(E) (*n* = 27), *eat*(A)*v* (*n* = 22), *lsa*(E)*-lsa*(A)*-eat*(A)*v* (*n* = 2), and *lsa*(E)*-eat*(A)*v* (*n* = 17) (Table 2).

**TABLE 2:** Quinupristin-dalfopristin (QDA) resistance gene profiles and ST types in *E. faecium* strains isolated from patients.

**Quinupristin-dalfopristin (QDA) resistance gene profiles**	**Number of isolates**	**QDA phenotype**			**ST types (No. of isolates)**	**Clonal complex (No. of isolates)**
		**R**	**I**	**S**		
*lsa(E)*	27	3	22	2	ST78 (23) ST18 (3) ST17 (1)	CC17 (27)
*lsa(E)- eat(A)v*	17	0	16	1	ST78 (13) ST571 (2) ST30 (1) ST414 (1)	CC17 (16) CC293 (1)
*lsa(E)- lsa(A)- eat(A)v*	2	2	0	0	ST747 (1) ST923 (1)	CC17 (1) Singleton (1)
*eat(A)v*	22	4	15	3	ST78 (14) ST812 (4) ST341 (2) ST94 (1) ST414 (1)	CC17 (17) CC39 (4) CC94 (1)
*no QDA resistance gene*	28	0	0	28	ST78 (14) ST18 (4) ST922 (3) ST812 (2) ST17 (1) ST389 (1) ST564 (1) ST921 (1) ST923 (1)	CC17 (26) CC39 (2)

QDA resistance was observed in 9 isolates (9.37%; 9/96), while 53 isolates (55.2%; 53/96) showed intermediate susceptibility. The majority of the *lsa*(E)-carrying strains (43/46; 93.48%) showed QDA resistance or an intermediate susceptible phenotype. Among the *lsa*(E)-positive strains, two strains carrying *lsa*(E)*-lsa*(A)*-eat*(A)*v* showed high QDA MIC values of 24 and 6 mg/L.

### Molecular Characterisation of *E. faecium* Isolates

MLST for all the isolates revealed fifteen ST types that belonged to four clonal complexes and one singleton (Table 2). ST78 (CC17) was the most frequent ST type and was identified in 64 of 96 isolates (64/96, 66.7%), followed by ST18 (CC17) (7/96, 7.3%) and ST812 (CC39) (6/96, 6.3%). Moreover, forty-four *lsa*(E)-positive strains (44/46, 95.6%) belonged to CC17.

### Conjugative Transfer of *lsa*(E) From *E. faecium* to *S. aureus*

**FIGURE 1:**
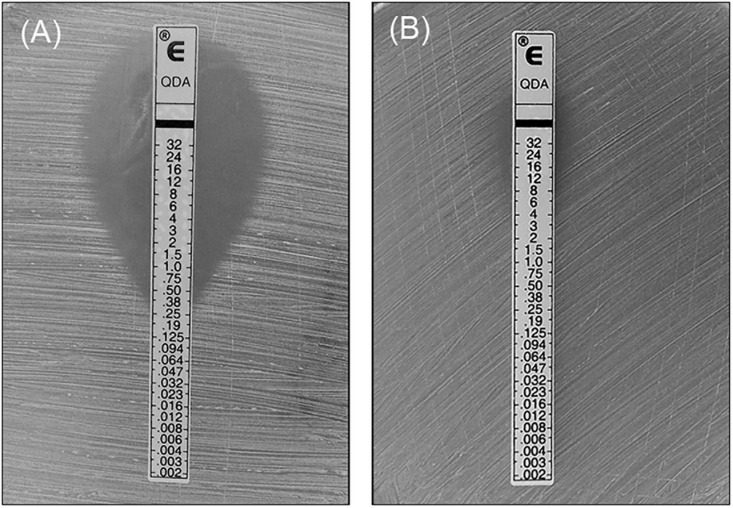
The MIC value of QDA against the recipient strain and the transconjugant strain. **(A)** Recipient strain, R3645; **(B)** Transconjugant strain, N7435/R3645.

Among the 40 mating tests performed, QDA resistance was successfully transferred in one conjugation at a frequency of 1.125 × 10^–7^ transconjugants per donor. Transfer occurred from *E. faecium* N7435 to *S. aureus* R3645. The match of the conjugated N7435-R3645 with the recipient was confirmed by comparing their *Sma*I-PFGE profiles (Supplementary Figure 1). One extra ∼100 kb band was observed in the N7435-R3645 *Sma*I-PFGE profile. The QDA resistance of conjugated N7435-R3645 was expressed at a higher level (MIC = 16 mg/L) than that of the parent *S. aureus* strain (MIC = 0.38 mg/L) (Figure 1). The erythromycin resistance of conjugated N7435-R3645 was also expressed at a higher level (MIC > 2048 mg/L) than that of the parent *S. aureus* strain (MIC = 512 mg/L). There was no difference in the MIC values of the other 17 antibiotics.

### Location and Stability of the *lsa*(E) Gene in the Transconjugant

**FIGURE 2:**
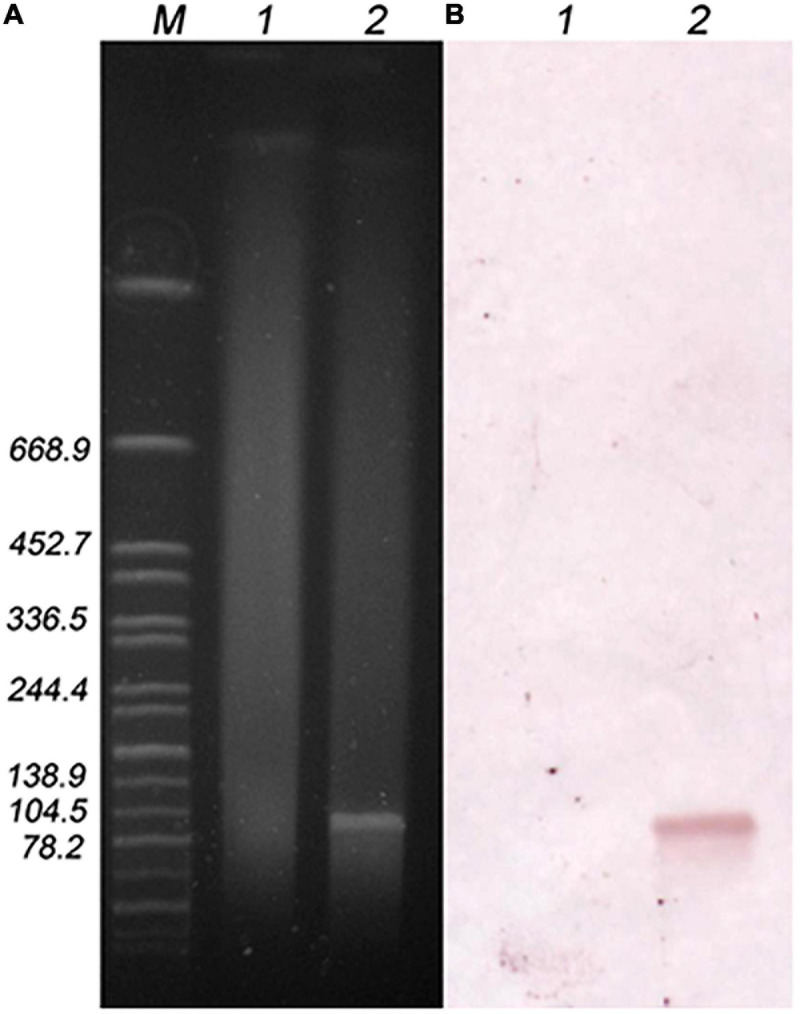
*S1*-PFGE plasmid profile of the recipient and transconjugant strains **(A)** and hybridisation with the *lsa*(E) probe **(B)**. M, Global reference strain H9812 of the *Salmonella* Braenderup serotype digested with the *Xba*I enzyme; lane 1, recipient strain (R3645); lane 2, transconjugant strain (N7435/R3645).

The location of the *lsa*(E) gene in the transconjugant N7435-R3645 was investigated by *S1*-PFGE followed by Southern blotting (Figure 2). *S1*-PFGE revealed that recipient R3645 did not harbour plasmids, while transconjugant N7435-R3645 carried a single ∼100 kb plasmid. Hybridisation assays showed that *lsa*(E) was located on the ∼100 kb plasmid in the transconjugant N7435-R3645.

*lsa*(E) was stable after ten overnight passages on antibiotic-free blood agar. The transconjugant continued to grow on MH agar supplemented with virginiamycin (4 and 8 mg/L) even after storage at 4 and −80°C for 4 weeks.

### Characteristics of the Transconjugated Plasmid

**FIGURE 3:**
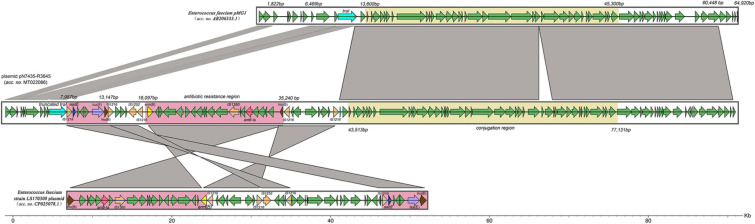
Structure of the *lsa*(E)-carrying plasmid pN7435-R3645 and similar regions in plasmid pMG1 and plasmid LS170308 of *E. faecium*. The structure of the plasmid pN7435-R3645 is illustrated based on the nucleotide sequences deposited in the GenBank database (MT022086). ORFs are indicated by arrows coloured as follows: orange, insertion sequences; purple, *lsa*(E); blue, *aadE*; pink, *ant6*-Ia; brown, *lnu*(B); yellow, *erm*(B); light blue, *traI* or truncated *traI*; and green, other genes. The similarities in the structures are indicated by grey shading. Antibiotic resistance regions and conjugation regions are shown in pink and yellow, respectively.

Next-generation sequencing (NGS) analysis of the pN7435-R3645 transconjugant showed that the complete sequence of the *lsa*(E)-carrying plasmid pN7435-R3645 was 92,396 bp in size and had a G + C content of 33% (accession no. MT022086). Sequence analysis identified 119 ORFs. The genetic map of pN7435-R3645 is shown in Figure 3 along with the maps of elements showing high nucleotide similarity and the main ORF features with two plasmids, *E. faecium* pMG1 (AB206333.1) and *E. faecium* LS170308 plasmid (CP025078.1).

pN7435-R3645 is highly equivalent to the 1–60,447 bp region of *E. faecium* pMG1, and this region of similarity is divided into two parts by the insertion of the *E. faecium* LS170308 plasmid. The insertion site was in the ORF region of the TraI topoisomerase-encoding gene. The pN7435-R3645 plasmid retained most of the genes from the *E. faecium* pMG1 plasmid and lost mainly the 1,822–6,469 bp and 60,448–64,920 bp regions, corresponding to the aminoglycoside resistance gene (*aac/aph*) and insertion sequence (IS) elements, respectively.

The conjugation region (43,513–77,131 bp; G + C content, 32.07%) of the pN7435-R3645 plasmid is approximately 33.6 kb. This region showed 99% identity with the conjugation region of pMG1 (13,600–45,300 bp). The *rep* gene of pN7435-R3645 showed 100% identity with that of pMG1, which did not belong to the *rep*1-19 family but belonged to a unique *rep* family. The IS elements in the pN7435-R3645 plasmid involved in possible recombination processes included mainly the *IS1216* transposase.

The resistance genes on the pN7435-R3645 plasmid were located mainly in the region of similarity with the *E. faecium* LS170308 plasmid. In addition, the plasmid structure was rearranged in these regions. Different AR elements were found in the following two pN7435-R3645 regions:

*lsa*(E) region (7,987–13,147 bp; G + C content, 35.69%). This region included three AR genes, *lsa*(E), *aadE* and *lnu*(B), which confer resistance to lincosamides/streptogramin A/pleuromutilins, aminoglycosides, and lincosamide, respectively. This region exhibited more than 99% nucleotide identity with multiple plasmids of *E. faecium* (plasmids of *E. faecium* strain LS170308, pEF37BA, pXD5, pY13, *etc.*), *E. faecalis* (pEF418, pE15, p11-27, *etc.*), *E. gallinarum* (pY15), and *S. aureus* (pV7037) as well as the chromosome region of *Streptococcus agalactiae.* This region was flanked by two identical IS1216 transposase genes with the same orientation.

*erm*(B), *ant6*-Ia, and *lnu*(B) regions (18,097–35,240 bp; G + C content, 35.72%). This segment contained three AR genes, *erm*(B), *ant6*-Ia, and *lnu*(B), which confer resistance to macrolides, aminoglycosides and lincosamide, respectively. This region exhibited 99% nucleotide identity with part of the *E. faecium* strain LS170308 plasmid and was flanked by two identical *IS1216* transposase genes.

## Discussion

Plasmids harbour a number of antibiotic genes and are widely found in enterococci, mainly *E. faecalis* and *E. faecium*, which are currently leading causes of multiresistant hospital-acquired infections. Conjugation is a primary means of intercellular DNA transfer in enterococci. Moreover, enterococci are reservoirs for antibiotic resistance genes, which can spread to other important pathogens, most notably *S. aureus*.

The present study provided the first evidence of the ability of the *lsa*(E) gene to undergo plasmid-mediated transfer and of the ability of an *E. faecium* plasmid carrying a *lsa*(E) gene to replicate in a clinical MRSA strain. To date, only the *van*(A) gene has been shown to be transferred from *E. faecalis* to *S. aureus* by conjugation *in vitro* (de Niederhausern et al., 2011). Peptide sex pheromones secreted by *S. aureus* induce conjugation-related mating functions and may play an important role in Tn*1546*-containing pheromone-responding plasmid transfer in *E. faecalis* (Showsh et al., 2001). To our knowledge, *lsa* (E) is the first gene that has been confirmed to be transferred from *E. faecium* to *S. aureus in vitro* and probably has a different transfer mechanism than the *van*(A) gene. Plasmid pN7435-R3645 in this study retained most of the genes in the *E. faecium* pMG1 plasmid. pMG1, which has been completely sequenced, is a 65 kb conjugative plasmid from *E. faecium* containing a Tn*4001*-like element and is a non-pheromone-responding plasmid (Ike et al., 1998; Tanimoto and Ike, 2008). It can transfer relatively well to other *E. faecium* strains in broth as well as to *E. faecalis* and *E. hirae*. pMG1 family elements have significantly contributed to the spread of vancomycin and gentamicin resistance among enterococci, particularly within *E. faecium* (Tomita et al., 2003). Although insertion of the plasmid pLS170308 region resulted in partial deletion of the TraI topoisomerase-encoding gene, pN7435-R3645 still retained the complete conjugation region of pMG1 (13,600–45,300 bp) (Tanimoto and Ike, 2008). Therefore, we speculated that the horizontal transfer of *lsa*(E) between *E. faecium* and *S. aureus* was dependent mainly on a non-pheromone-responding pMG1-like plasmid through conjugation.

Another *lsa*(E)-carrying non-conjugative plasmid, pY13 (28,489 bp), from a porcine linezolid-resistant *E. faecium* isolate has been reported (Si et al., 2015). The conjugative plasmid pN7435-R3645, approximately 92,396 bp, in this study is much larger than pY13 and has a structure different from that of pY13. Rearrangement and inversion regions were observed on the plasmid pN7435-R3645. Since all of these segments were flanked by ISs, pN7435-R3645 may have derived from interplasmidic recombination events in which ISs, such as IS*1216* and IS*1252*, were involved.

In the present study, four clinical ST239 MRSA strains were selected as recipients, and only one strain (R3645) was successfully transferred. Mutations in genes of the SauI type I restriction-modification (RM) system and deficiency in the type IV RM system have been shown to increase a strain’s ability to accept foreign DNA (Waldron and Lindsay, 2006; Corvaglia et al., 2010). Intact type I and IV RM systems were found in R3645 (data not shown). Other characteristics, such as mutations of CRISPR loci, that may contribute to the ability to acquire *lsa*(E)-carrying non-conjugative plasmids need to be further investigated.

*E. faecalis* is intrinsically resistant to QDA as a result of the presence of the *lsa* determinant, while *E. faecium* always acquires QDA resistance (Singh et al., 2002). To date, the prevalence of QDA resistance among *E. faecium* clinical isolates in many countries has been low, but relatively high resistance rates have occasionally been reported, such as 6.7% (9/135) in Poland (Sadowy et al., 2013), 10% (25/249) in Korea (Oh et al., 2005), and 60% in northwest Iran (6/10) (Haghi et al., 2019). The rate of intermediate resistance to QDA is relatively high in some countries, such as 17.6% (28/159) in Japan (Isogai et al., 2013), 26.7% (36/135) in Poland (Sadowy et al., 2013) and 28.9% (250/865) in Greece (Karanika et al., 2008). An investigation in a Chinese hospital in Wenzhou reported that 9 of 911 (1.0%) *E. faecium* isolates were resistant to QDA (Wang et al., 2016). In this study, QDA resistance was observed in 9 isolates (9.37%; 9/96), while 53 isolates (55.2%; 53/96) showed intermediate susceptibility. This finding indicated that QDA resistance differed among hospitals and regions in China. Although QDA has not been marketed in China, virginiamycin, which belongs to the same antibiotic class as QDA, has been widely used as an animal growth promoter in poultry, cattle and swine. The resistance of *E. faecium* strains isolated from animals to QDA ranged from 2.2 to 33.6%, and 38.5–83.2% of the strains were classified as not sensitive in European countries from 2004 to 2014 (Wang et al., 2016; de Jong et al., 2019). However, virginiamycin has been banned for use as a growth promoter in Europe since 1999. This may be explained by the possible co-selection of resistance genes by compounds currently approved to treat clinical diseases.

A high prevalence of *lsa*(E) (47.92%, 46/96) was found among clinical *E. faecium* isolates, and the majority of *lsa*(E)-carrying strains (43/46; 93.48%) showed QDA resistance or an intermediate susceptible phenotype in this study. Acetyltransferases encoded by *vat*D and *vat*E have been found in enterococci from various sources, including humans, animals and the environment in Europe, the United States and Asia (Soltani et al., 2000; Werner et al., 2000; Jackson et al., 2007; Hwang et al., 2010); however, the *vatD* and *vatE* genes were not detected in this study. To date, only two papers have reported the distribution of *lsa*(E) in enterococcus, and both papers are from China. The *lsa*(E) gene was found in 30.3% (10/33) of human enterococcal strains and 53.6% (37/69) of swine enterococcal strains in Henan Province, China. Most of them were clonally unrelated, with the exception of *E. faecium* ST29 (*n* = 4) and ST362 (*n* = 4) (Li et al., 2014). The *lsa*(E) gene was also detected in five *E. faecalis* strains, one *E. faecium* strain and one *E. gallinarum* strain among thirty-five enterococcal strains isolated from a pig farm in Guangxi Province, China (Si et al., 2015). In this study, thirty-six *lsa*(E)-positive strains (36/46, 78.3%) belonged to ST78, which is an epidemic clone in hospitals in China (Sun et al., 2019). This result suggested that the high *lsa*(E) detection rate in clinical strains may be due to the spread of *E. faecium*-resistant clones.

In conclusion, a high prevalence of *lsa*(E) was found in clinical *E. faecium* strains. An *lsa*(E)-carrying plasmid that can be transferred from *E. faecium* to *S. aureus in vitro* by conjugation was identified. This MDR pMG1-like plasmid may act as a vector in the dissemination of antimicrobial resistance among species.

## Data Availability Statement

The datasets presented in this study can be found in online repositories. The names of the repository/repositories and accession number(s) can be found below: https://www.ncbi.nlm.nih.gov/genbank/, MT022086.

## Author Contributions

X-MY, JW, and J-ZZ conceived the study. X-MY wrote the manuscript and performed the Southern blotting experiment. JW, H-BJ, HY, and Y-HY collected the strains and performed the antibiotic resistance experiments. X-XT, F-LM, BZ, and YH carried out the molecular typing, mating, and QDA resistance gene detection. X-MY and Y-HY analysed the genome sequencing data. J-ZZ revised the manuscript. All authors read and approved the final manuscript.

## Conflict of Interest

The authors declare that the research was conducted in the absence of any commercial or financial relationships that could be construed as a potential conflict of interest.

## Footnotes

1 https://pubmlst.org/bigsdb?db=pubmlst_efaecium_seqdef

2 http://topaz.gatech.edu/

3 http://blast.ncbi.nlm.nih.gov/blast
