# Supplementary material for: A Conjugative MDR pMG1-Like Plasmid Carrying the lsa(E) Gene of Enterococcus faecium With Potential Transmission to Staphylococcus aureus
Source: Front Microbiol. 2021 Jun 4;12:667415. doi: 10.3389/fmicb.2021.667415 (PMC8212935; doi:10.3389/fmicb.2021.667415)
Supplement: Supplementary file 1 [file Data_Sheet_1.docx]

Supplementary Material

Table 1. The background of the 96 *E. faecium* strains

| Strain name | Sample type | Sex | Age(Year) |
| --- | --- | --- | --- |
| N9800 | Bile | Female | 63 |
| P9772 | Bile | Female | 79 |
| R2935 | Bile | Female | 80 |
| N9054 | Secretion | Female | 61 |
| R6783 | Ascites | Female | 57 |
| P8798 | Ascites | Female | 81 |
| R5267 | Urine | Female | 46 |
| P3030 | Urine | Female | 51 |
| R8168 | Urine | Female | 57 |
| N8064 | Urine | Female | 62 |
| N1876 | Urine | Female | 67 |
| R3104 | Urine | Female | 68 |
| P5801 | Urine | Female | 71 |
| P7952 | Urine | Female | 74 |
| N9882 | Urine | Female | 77 |
| P7621 | Urine | Female | 77 |
| N7435 | Urine | Female | 81 |
| N7331 | Urine | Female | 89 |
| N9673 | Urine | Female | 89 |
| P1485 | Urine | Female | 89 |
| N9761 | Urine | Female | 94 |
| R31 | Urine | Female | 106 |
| R5398 | Urine | Female | 106 |
| R7913 | Urine | Female | 106 |
| P2700 | Sputum | Female | 40 |
| N2201 | Sputum | Female | 53 |
| N7770 | Sputum | Female | 55 |
| N9349 | Sputum | Female | 66 |
| N7692 | Sputum | Female | 67 |
| R1238 | Sputum | Female | 73 |
| P84 | Sputum | Female | 80 |
| N2247 | Sputum | Female | 93 |
| 3688 | Blood | Female | 40 |
| 7672 | Blood | Female | 52 |
| 3240 | Blood | Female | 57 |
| 9943 | Blood | Female | 63 |
| 9816 | Blood | Female | 63 |
| 688 | Blood | Female | 63 |
| 5659 | Blood | Female | 71 |
| 6354 | Blood | Female | 75 |
| 6474 | Blood | Female | 75 |
| N2456 | Blood | Female | 94 |
| N3427 | Blood | Female | 94 |
| P2738 | Throat swab | Female | 75 |
| 9200 | Drainage | Female | 52 |
| P3673 | Drainage | Female | 61 |
| R375 | Drainage | Female | 80 |
| P9222 | Rectal swab | Female | 93 |
| R6715 | Rectal swab | Female | 94 |
| P1407 | Central venous catheter | Female | 55 |
| P8501 | Central venous catheter | Female | 90 |
| R1805 | Secretion | Male | 75 |
| N9014 | Secretion | Male | 88 |
| P9672 | Ascites | Male | 53 |
| 4103 | Ascites | Male | 73 |
| N1384 | Ascites | Male | 78 |
| N1591 | Urine | Male | 24 |
| R8067 | Urine | Male | 29 |
| P9940 | Urine | Male | 43 |
| N1457 | Urine | Male | 59 |
| P2041 | Urine | Male | 63 |
| P3642 | Urine | Male | 63 |
| P7139 | Urine | Male | 68 |
| R6852 | Urine | Male | 74 |
| N7091 | Urine | Male | 74 |
| P3784 | Urine | Male | 74 |
| P604 | Urine | Male | 75 |
| P4366 | Urine | Male | 76 |
| P3814 | Urine | Male | 76 |
| P480 | Urine | Male | 77 |
| P8786 | Urine | Male | 77 |
| N136 | Urine | Male | 79 |
| P5238 | Urine | Male | 79 |
| N9696 | Urine | Male | 82 |
| R4989 | Urine | Male | 84 |
| R5520 | Urine | Male | 84 |
| R5602 | Urine | Male | 84 |
| N3095 | Urine | Male | 85 |
| N2142 | Urine | Male | 85 |
| N6570 | Urine | Male | 85 |
| P6691 | Urine | Male | 87 |
| N7395 | Pus | Male | 70 |
| N7428 | Pus | Male | 82 |
| P563 | Sputum | Male | 65 |
| N130 | Sputum | Male | 77 |
| N3572 | Sputum | Male | 77 |
| N7675 | Sputum | Male | 86 |
| 1670 | Blood | Male | 52 |
| N119 | Blood | Male | 67 |
| 9472 | Blood | Male | 72 |
| 284 | Blood | Male | 72 |
| 5118 | Blood | Male | 79 |
| 8819 | Blood | Male | 13 Days |
| N9065 | Vaginal swab | Male | 38 |
| 8525 | Drainage | Male | 67 |
| P3726 | Drainage | Male | 74 |
| P2505 | Drainage | Male | 76 |
| P2125 | Central venous catheter | Male | 88 |

Table S2. Primer sequences used for the detection of quinupristin/dalfopristin resistance genes

| Primer | Sequence (5’→3’) | Reference |
| --- | --- | --- |
| *lsa*(A)*-F* | TGGTCGATGAACCGTGAAGG | The present study |
| *lsa*(A)*-R* | TCAATCACTAGCATTGCAGGC |  |
| *lsa (C)-F* | GGCTATGTAAAACCTGTATTTG | (Si et al., 2015) |
| *lsa (C)-R* | ACTGACAATTTTTCTTCCGT |  |
| *lsa(E)-F* | ACGGACGCGGTAAAACTACT | The present study |
| *lsa(E)-R* | AGGACCTTCGTTTGCTCACC |  |
| *vatD-F* | TGGGTCCGAATCCTATGAAAATGT | The present study |
| *vatD-R* | TCCCCGATTTTTACTCCTGGC |  |
| *vatE-F* | ATCATGAACGGTGCCAACCA | The present study |
| *vatE-R* | TTTTGCCCAAACCACACGTC |  |
| *vatH-F* | GTGGGAAAAGCATACACCT | (Jung et al., 2010) |
| *vatH-R* | TTGCAGGATTACCACCAAC |  |
| *vgaD-F* | CAACTGGAGCGAGCTGTTA | (Jung et al., 2010)2 |
| *vgaD-R* | GACAGCCGGATAATCTTTTG |  |
| *eat*(A)V-F | TTTGAACAACCTCCGAAAGC | (Isnard et al., 2013) |
| *eat*(A)V-R | TTTCTGTGCCTGCATCTGTC |  |

**References:**

Isnard, C., Malbruny, B., Leclercq, R., and Cattoir, V. (2013). Genetic basis for in vitro and in vivo resistance to lincosamides, streptogramins A, and pleuromutilins (LSAP phenotype) in *Enterococcus faecium*. *Antimicrob. Agents. Chemother. 57*, 4463-4469.

Jung, Y.H., Shin, E.S., Kim, O., Yoo, J.S., Lee, K.M., Yoo, J.I., et al. (2010). Characterization of two newly identified genes, *vgaD* and *vatH*, [corrected] conferring resistance to streptogramin A in *Enterococcus faecium.* *Antimicrob. Agents. Chemother. 54*, 4744-4749.

Si, H., Zhang, W.J., Chu, S., Wang, X.M., Dai, L., Hua, X., et al. (2015). Novel plasmid-borne multidrug resistance gene cluster including *lsa*(E) from a linezolid-resistant *Enterococcus faecium* isolate of swine origin. *Antimicrob. Agents. Chemother. 59*, 7113-7116.

**
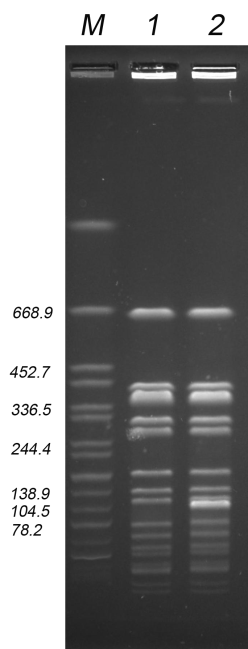
**

**Supplementary Figure 1.** *SmalI*-PFGE of the recipient and transconjugant strains. Lane 1, recipient strain (R3645); lane 2, transconjugant strain (N7435/R3645).
